# Molecular evolution of virulence genes and non-virulence genes in clinical, natural and artificial environmental *Legionella pneumophila* isolates

**DOI:** 10.7717/peerj.4114

**Published:** 2017-12-04

**Authors:** Xiao-Yong Zhan, Qing-Yi Zhu

**Affiliations:** 1 Guangzhou KingMed Center for Clinical Laboratory, Guangzhou, China; 2 KingMed School of Laboratory Medicine, Guangzhou Medical University, Guangzhou, China; 3 The First Affiliated Hospital, Sun Yat-Sen University, Guangzhou, China

**Keywords:** *Legionella pneumophila*, Non-virulence genes, Virulence genes, Molecular phylogeny, Genetic diversity, Molecular evolution

## Abstract

**Background:**

*L. pneumophila* is the main causative agent of Legionnaires’ disease. Free-living amoeba in natural aquatic environments is the reservoir and shelter for *L. pneumophila.* From natural water sources,* L. pneumophila* can colonize artificial environments such as cooling towers and hot-water systems, and then spread in aerosols, infecting the susceptible person. Therefore, molecular phylogeny and genetic variability of *L. pneumophila* from different sources (natural water, artificial water, and human lung tissue) might be distinct because of the selection pressure in different environments. Several studies researched genetic differences between *L. pneumophila* clinical isolates and environmental isolates at the nucleotide sequence level. These reports mainly focused on the analysis of virulence genes, and rarely distinguished artificial and natural isolates.

**Methods:**

We have used 139* L. pneumophila* isolates to study their genetic variability and molecular phylogeny. These isolates include 51 artificial isolates, 59 natural isolates, and 29 clinical isolates. The nucleotide sequences of two representative non-virulence (NV) genes (*trpA, cca*) and three representative virulence genes (*icmK, lspE, lssD*) were obtained using PCR and DNA sequencing and were analyzed.

**Results:**

Levels of genetic variability including haplotypes, haplotype diversity, nucleotide diversity, nucleotide difference and the total number of mutations in the virulence loci were higher in the natural isolates. In contrast, levels of genetic variability including polymorphic sites, theta from polymorphic sites and the total number of mutations in the NV loci were higher in clinical isolates. A phylogenetic analysis of each individual gene tree showed three to six main groups, but not comprising the same *L. pneumophila* isolates. We detected recombination events in every virulence loci of natural isolates, but only detected them in the *cca* locus of clinical isolates. Neutrality tests showed that variations in the virulence genes of clinical and environmental isolates were under neutral evolution. T*rpA* and *cca* loci of clinical isolates showed significantly negative values of Tajima’s D, Fu and Li’s D* and F*, suggesting the presence of negative selection in NV genes of clinical isolates.

**Discussion:**

Our findings****reinforced the point that the natural environments were the primary training place for *L. pneumophila* virulence, and intragenic recombination was an important strategy in the adaptive evolution of virulence gene. Our study also suggested the selection pressure had unevenly affected these genes and contributed to the different evolutionary patterns existed between NV genes and virulence genes. This work provides clues for future work on population-level and genetics-level questions about ecology and molecular evolution of *L. pneumophila,* as well as genetic differences of NV genes and virulence genes between this host-range pathogen with different lifestyles.

## Introduction

*Legionella pneumophila* (*L. pneumophila*) is the main causative agent of Legionnaires’ disease (Fields, Benson & Besser, 2002; Gomez-Valero et al., 2014). It is a gram-negative bacterium in natural water environments such as pools, rivers and lakes, as well as in various artificial water systems worldwide (Gomez-Valero, Rusniok & Buchrieser, 2009). Free-living amoeba in natural aquatic environments is the reservoir and shelter for *L.* *pneumophila.* From natural environments, it can colonize artificial water environments such as cooling towers and hot-water systems, and then spread in aerosols, infecting the susceptible person (Escoll et al., 2013; Lau & Ashbolt, 2009). It is clear the intra-amoebal lifestyle of *L. pneumophila* in natural environments is important to *L. pneumophila* genome*.* It provides the primary evolutionary pressure to this microorganism and shapes the genomic structure of this bacterium. In this process, amoeba may act as a gene smelter, allowing diverse microorganisms to evolve by gene acquisition and loss, and then makes *L. pneumophila* better adapt to the intra-amoebal lifestyle or evolve into new pathogenic forms (Gomez-Valero et al., 2011; Khodr et al., 2016; Richards et al., 2013; Zhan, Hu & Zhu, 2016). Within *L. pneumophila* lifestyles, artificial environments are an intermediate post for this microorganism from the natural environments to human. Since person-to-person transmission of *L. pneumophila* has rarely been reported, the infection of human lung tissue may be an evolutive end for *L. pneumophila* (Correia et al., 2016; Costa et al., 2014). Therefore, the three environments (natural water, artificial water, and human lung tissue) where *L. pneumophila* inhabits, play different roles in its life and may influence the shape of *L. pneumophila* genome. Several reports proved that *L. pneumophila* clinical isolates displayed less genetic diversity than environmental isolates (Coscolla & Gonzalez-Candelas, 2009; Costa et al., 2012; Costa et al., 2014). These results could illustrate that at the evolutive end, there is no more selective pressure to shape the *L. pneumophila* genome. Another explanation is that isolates of *L. pneumophila* recovered from clinical cases are a limited and specific subset of all genotypes existing in nature, and they may be an especially adapted group of clones (Coscolla & Gonzalez-Candelas, 2009). However, these reports focused on the analysis of virulence-related genes, and rarely distinguished artificial and natural isolates (Coscolla & Gonzalez-Candelas, 2009; Costa et al., 2012; Costa et al., 2014).

Our goal was to determine the genetic diversity and population structure of *L. pneumophila* isolates from different sources at none-virulence (NV) gene and virulence gene levels, respectively, and to identify the molecular mechanisms operating in the evolution of these genes. We have studied the genetic and population diversity by comparing nucleotide sequences from five representative gene loci. These loci included two NV loci which were common among a set of bacterial genomes (tryptophan synthase α subunit-encoding gene, *trpA* and tRNA nucleotidyltransferase gene, *cca*), and three virulence loci (*icmK, lspE,* and *lssD*). These nucleotide sequences were from 51 artificial isolates, 59 natural isolates and 29 clinical or disease-related isolates. The *trpA* gene controls the sequence of reactions from chorismic acid to tryptophan, and the *cca* gene catalyzes the accurate synthesis of the -C-C-A terminus of *tRNA* (Dounce, Morrison & Monty, 1955; Gebran et al., 1994). They all play fundamental roles in the survival of bacteria**.** The virulence genes belong to different protein secretion systems, including the Dot/Icm type IVB protein secretion system (*icmK*), the Lsp type II secretion system (*lspE*), and the type I Lss secretion system(*lssD*). They represent key virulence and play crucial roles in ecology and pathogenesis of *L. pneumophila* (De Buck, Anne & Lammertyn, 2007; Fuche et al., 2015; Korotkov, Sandkvist & Hol, 2012).

Our results showed the levels of genetic variability in the virulence loci were higher in natural isolates. In contrast, levels of genetic variability in the NV loci were higher in clinical isolates. Molecular phylogeny of these genes showed three to six main groups, but none of them contained the same origin of *L. pneumophila* isolates. Intragenic recombination of *cca*, *lssD*, *lspE* and *icmK* genes was also detected in this study. It was favored as an important evolutionary mechanism for natural and clinical isolates by influencing the population genetic structure of *L. pneumophila.*

## Materials and Methods

### *L. pneumophila* isolates

One hundred and thirty-nine strains of *L. pneumophila* were enrolled in this study. These isolates included 51 artificial isolates, 59 natural isolates, and 29 clinical isolates. The source natures, geographic locations, collection dates and sequence types (STs) based on the Sequence-Based Typing (SBT) scheme (Gaia et al., 2005; Ratzow et al., 2007) of these isolates are shown in Table S1. Briefly, the 29 clinical strains were isolated between 1947 and 2012, from the non-China regions, including different cities of USA, Germany, UK, France, etc. Their details were obtained from the NCBI database (https://www.ncbi.nlm.nih.gov). The environmental isolates were from 14 different sites in two cities (Guangzhou and Jiangmen) of Guangdong Province, China between October 2003 and September 2007. All the environmental isolates were selected for sequencing partial *trpA, cca , lssD, lspE,* and *icmK* genes. We selected the most variable regions of these genes through a sequence alignment with the known sequences in the NCBI database, in order to achieve maximum genetic variability. The variability of the gene regions was measured by calculating the single-nucleotide polymorphisms of the sequence using Dnasp v5 (Librado & Rozas, 2009; Rozas, 2009) (Table S2).

### Genomic DNA extraction, PCR, and DNA sequencing

Genomic DNA extraction of the artificial and natural strains was performed as shown in our previous report (Zhan, Hu & Zhu, 2016). PCR was employed to amplify fragments of DNA. The corresponding oligonucleotide primers are shown in Table S2. The PCR was performed using an EasyPfu PCR SuperMix (Transgene Biotech, Beijing, China) according to the manufacturer’s instructions and carried out using the GeneAmp PCR system (MJ Research PTC-200) with the following thermal conditions: 95 °C for 3 min followed by 35 cycles of 95 °C for 20 s, 60 °C for 20 s and 72 °C for 30 s (*lspE*, *lssD,* and *icmK* loci) or 70 s (*cca* and *trpA* loci), and a final extension at 72 °C for 5 min. For confirmation purposes, each PCR reaction was performed with a positive control (*L. pneumophila* strain ATCC33152 genomic DNA as the PCR template) and a negative control (sterile water as the PCR template). PCR products were purified using an EasyPure Quick Gel Extraction (Transgene Biotech, Beijing, China) and then transferred to Guangzhou IGE Biotechnology Ltd for sequencing.

### Sequence analysis

The quality of DNA sequencing was manually checked by Chromas (http://technelysium.com.au). The corresponding gene sequences of 29 clinical isolates were obtained from the NCBI database. Multiple sequence alignments were performed using ClustalX 2.1 (Chenna et al., 2003; Thompson et al., 1997). Genetic variability analyses were performed using DnaSP v5 (Librado & Rozas, 2009; Rozas, 2009). Phylogenetic analyses were conducted by a MEGA7 package (Kumar, Stecher & Tamura, 2016). Neighbor-Joining (NJ) phylogenetic trees were obtained for each locus separately with the MEGA7 based on the Kimura 2-parameter model (Kimura, 1980; Saitou & Nei, 1987). The tree was drawn to scale, with branch lengths in the same units as those of the evolutionary distances used to infer the phylogenetic tree. NJ tree nodes were evaluated by bootstrapping with 1,000 replicates. The ratios of synonymous and non-synonymous substitutions were calculated according to the Nei-Gojobori method with Jukes-Cantor correction as implemented in the MEGA7 (Kumar, Stecher & Tamura, 2016).

### Molecular evolution analysis

The aligned sequences of the five loci were screened using RDP4 to detect intragenic recombination (Martin et al., 2015; Martin et al., 2017). Six methods implemented in the program RDP4 were utilized. These methods were RDP (Martin & Rybicki, 2000), GENECONV (Padidam, Sawyer & Fauquet, 1999), BootScan (Martin et al., 2005), MaxChi (Smith, 1992), Chimaera (Posada, 2002), and SiScan (Gibbs, Armstrong & Gibbs, 2000). Potential recombination event was considered as that identified by at least two methods according to Coscolla’s report (Coscolla & Gonzalez-Candelas, 2009). Common settings for all methods were to consider sequences as linear, statistical significance was set at the *P* < 0.05 level, with Bonferroni correction for multiple comparisons and requiring phylogenetic evidence and polishing of breakpoints.

Tajima’s D, Fu and Li’s D* and F* were calculated for testing the mutation neutrality hypothesis as previously described by Coscolla and colleagues (2007). These statistics were calculated with the program Dnasp v5 (Rozas, 2009) using a statistical significance level *P* < 0.05 and applying the false discovery rate to correct for multiple comparisons, and 1000 replicates in a coalescent simulation. Non-neutrality evolution was considered as that identified by at least two of the methods.

### Population structure analysis

Hierarchical analysis of molecular variance (AMOVA) for clinical and environmental sequences using the alignment of the five loci was performed using Arlequin Ver3.5.2 (Excoffier & Lischer, 2010). This analysis provides estimates of variance components and F-statistics analogues speculating the correlation of haplotype diversity at different levels of the hierarchical subdivision. We defined the hierarchical subdivision of these isolates at three levels. At the upper level, the three groups considered were clinical, artificial and natural isolates. As populations within groups, the intermediate level, we reckoned the strains isolated from the same geographic location as subpopulations. Therefore, natural and artificial isolates were both split into two subgroups based on the cities where they were isolated (Guangzhou and Jiangmen subgroups), and clinical strains were also divided into two subgroups based on the continents where they were isolated (eg. America or non-America, including Europe and Australia). The third level corresponded to the different haplotypes which were found within the six subgroups considered in the previous level. The statistical significance of fixation indices was tested using a non-parametric permutation approach (Excoffier, Smouse & Quattro, 1992).

### Nucleotide sequence accession numbers

The 550 sequences from *L. pneumophila* environmental isolates determined in this study were deposited in the GenBank Nucleotide Sequence Database with accession numbers KY708328 –KY708437 (*cca*), KY708438 –KY708547 (*trpA*), KY708768 –KY708877 (*lssD*), KY708658 –KY708767 (*lspE*) and KY708548 –KY708657 (*icmK*).

## Results

### Sequence analysis and genetic variability of *L. pneumophila* isolates from different sources

In general, we obtained the gene sequences from 29 clinical, 59 natural and 51 artificial environmental isolates. Genetic diversity estimates in all the clinical, natural and artificial isolates are presented in Table 1. The highest nucleotide diversity (*π*) in the *L .pneumophila* isolates was found in the *lssD* locus, varied from 0.04213 to 0.05399, while the lowest nucleotide diversity was found in *trpA* locus, varied from 0.01041 to 0.01246. Both the most haplotype (*h*) and highest haplotype diversity (*Hd*) of the five gene loci was found in natural isolates. For the *trpA* locus, the nucleotide diversity, number of polymorphic nucleotide sites (*S*), population mutation ration (*θ*), average number of pairwise nucleotide differences (*k*), and the total number of mutations (*η*) were all higher in clinical isolates. In contrast, another NV locus, *cca* did not show higher nucleotide diversity and nucleotide differences in clinical isolates, but the number of polymorphic nucleotide sites, population mutation ratio and the total number of mutations were higher in clinical isolates. The highest nucleotide diversity and the average number of pairwise nucleotide differences of *cca* locus were found in artificial isolates. Different results were found in the three virulence loci: the haplotype, haplotype diversity, nucleotide diversity and the average number of pairwise nucleotide differences were higher in natural isolates.

**Table 1:** Summary of genetic diversity analyses for the 5 gene loci in *L. pneumophila* clinical (C), artificial (A), and natural (N) environmental isolates.

Gene type	locus	Strain type	Sequence, (n)	Sequence length	*h*	*Hd*	SD of Hd	*π*	SD of *π*	*S*	*θ*	SD of *θ*	*k*	*η*	*dN/dS*
NV genes	*cca*	C	29	1082	10	0.692	0.092	0.01749	0.00542	**130**	**0.03059**	0.00268	18.921	**136**	0.1034
		N	59	1082	**16**	**0.892**	0.019	0.01935	0.00072	70	0.01392	0.00166	20.936	71	**0.1085**
		A	51	1082	6	0.660	0.062	**0.02212**	0.00439	121	0.02486	0.00226	**23.929**	124	0.0864
	*trpA*	C	29	748	9	0.690	0.091	**0.01216**	0.00486	**76**	**0.02587**	0.00297	**9.094**	**82**	0.0633
		N	59	748	**14**	**0.852**	0.026	0.01041	0.00066	44	0.01266	0.00191	7.786	47	**0.1001**
		A	51	748	7	0.590	0.075	0.01214	0.00374	58	0.01723	0.00226	9.078	62	0.0801
Virulence genes	*lssD*	C	29	330	7	0.640	0.092	0.04213	0.01229	63	**0.04861**	0.00612	13.901	68	**0.0502**
		N	59	330	**11**	**0.793**	0.039	**0.05399**	0.00932	**72**	0.04696	0.00553	**17.817**	**79**	0.0353
		A	51	330	4	0.404	0.082	0.04241	0.01122	71	0.04782	0.00568	13.994	77	0.0398
	*lspE*	C	29	331	7	0.608	0.100	0.02834	0.00571	37	0.02846	0.00468	9.379	39	0.0165
		N	59	331	**13**	**0.839**	0.031	**0.03748**	0.00223	49	0.03186	0.00455	**12.407**	53	**0.0378**
		A	51	331	8	0.696	0.064	0.02937	0.00608	**54**	**0.03626**	0.00493	9.721	**61**	0.0341
	*icmK*	C	29	385	9	0.690	0.091	0.03363	0.00647	65	0.04299	0.00533	12.948	71	0.0913
		N	59	385	**50**	**0.989**	0.008	**0.04775**	0.00217	**77**	0.04305	0.00491	**18.382**	**104**	**0.2543**
		A	51	385	37	0.978	0.011	0.03995	0.00655	76	**0.04387**	0.00503	15.380	91	0.1603

**Notes.**

*h* Haplotypes Hd Haplotype diversity *π* Nucleotide diversity *S* Polymorphic sites *θ* Theta (per site) from S, population mutation ration *k* Nucleotide differences *η* Total number of mutations

The rates of non-synonymous substitutions per non-synonymous site (*dN*) were extremely low and different between these genes, ranging from 0.00243 (for *lspE* in clinical isolates) to 0.0295 (for *icmK* in natural isolates), despite the relatively similar values of polymorphic sites (37 vs. 77). Synonymous substitutions (*dS)* ranged from 0.0338 for *trpA* in natural isolates to 0.3091 for *lssD* in natural isolates (Table S3). An obviously different *dN/dS* ratio was observed, ranging from 0.0165 (for *lssD* in clinical isolates) to 0.2543 (for *icmK* in natural isolates) (Table 1). We found different *dN/dS* ratios among clinical, natural, and artificial isolates. *dN/dS* ratios of the *cca, trpA, lspE,* and *icmK* locus were higher in natural isolates than in artificial and clinical isolates (Table 1).

**Figure 1:**
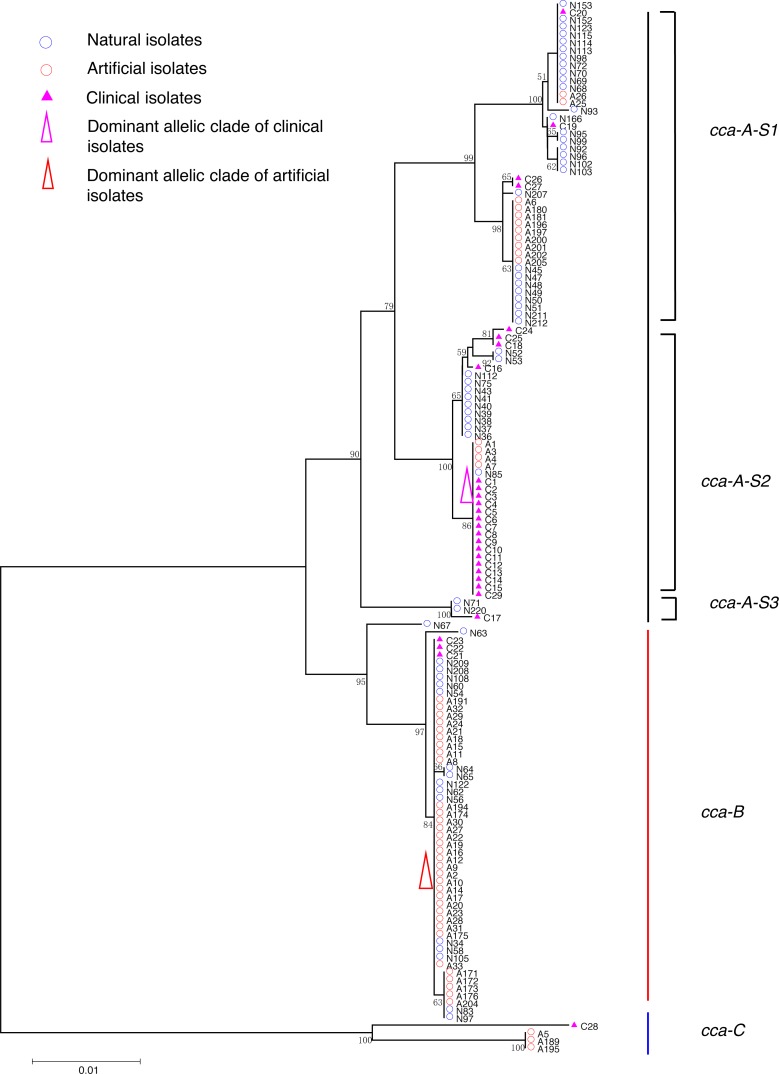
Neighbor-Joining tree of *L. pneumophila* isolates from DNA sequences of *cca* locus. Strain types, source natures and geographic locations of these isolates were shown in Table S1. Bootstrap support values (1,000 replicates) for nodes higher than 50% are indicated next to the corresponding node. Three main groups of the clades could be found.

**Figure 2:**
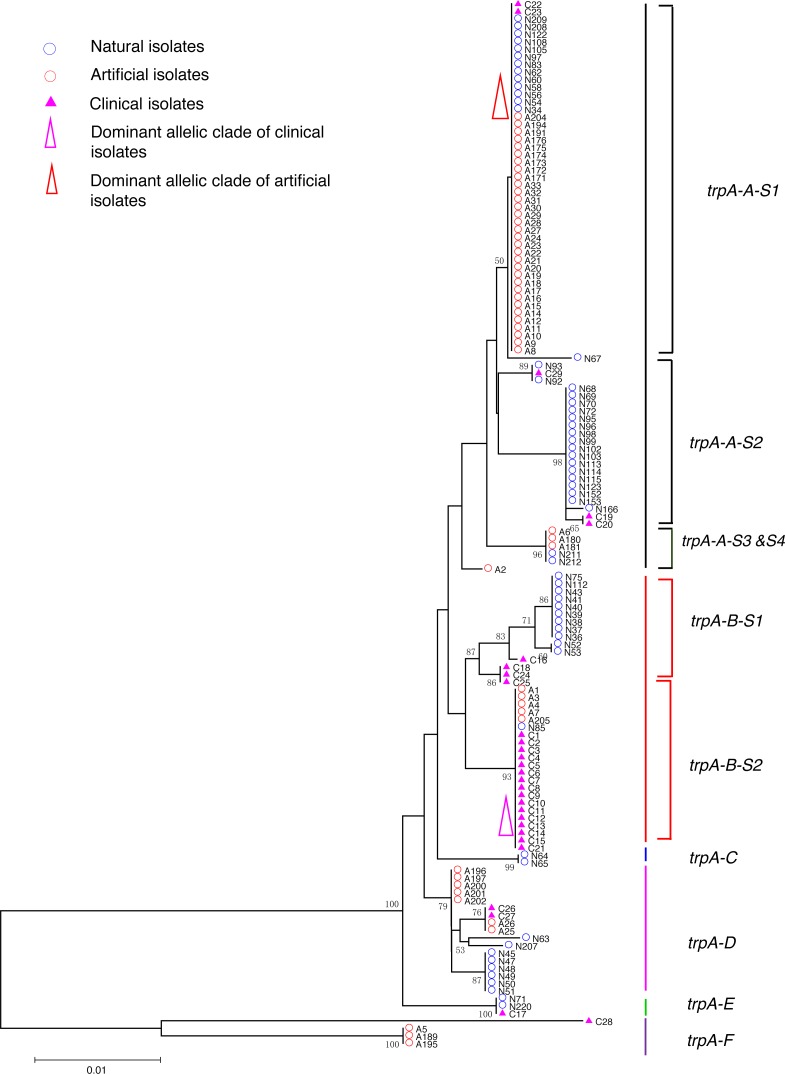
Neighbor-Joining tree of *L. pneumophila* isolates from DNA sequences of *trpA* locus. Strain types, source natures and geographic locations of these isolates were shown in Table S1. Bootstrap support values (1,000 replicates) for nodes higher than 50% are indicated next to the corresponding node. Six main groups of the clades could be found.

**Figure 3:**
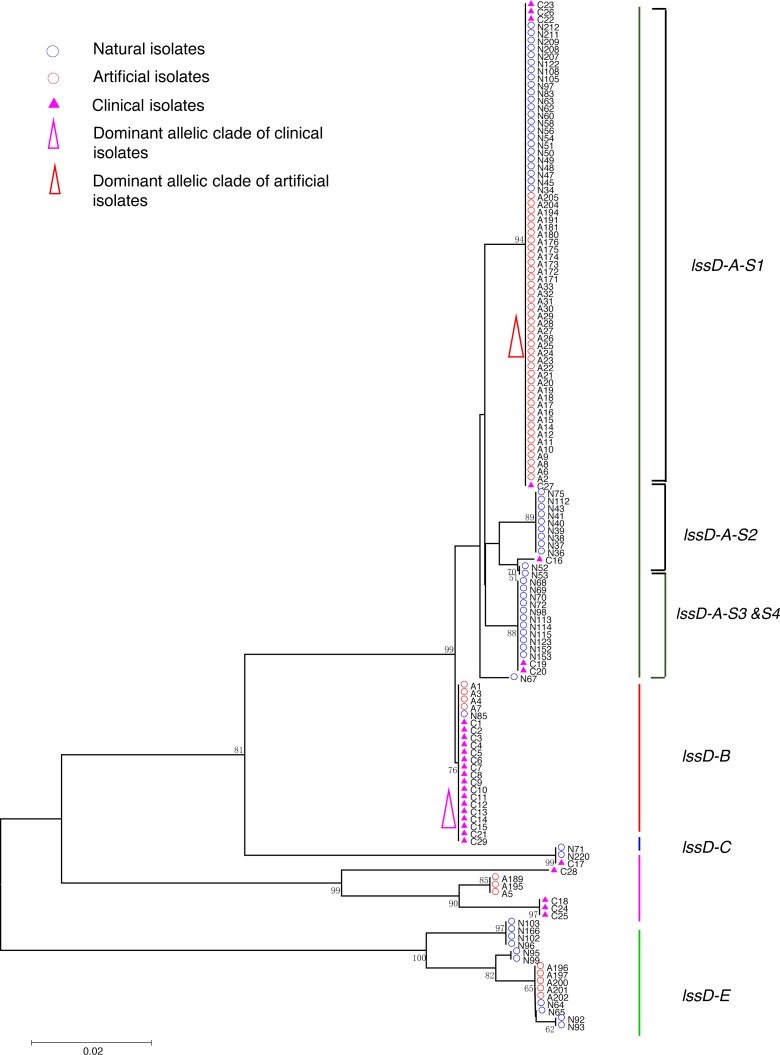
Neighbor-Joining tree of *L. pneumophila* isolates from DNA sequences of *lssD* locus. Strain types, source natures and geographic locations of these isolates were shown in Table S1. Bootstrap support values (1,000 replicates) for nodes higher than 50% are indicated next to the corresponding node. Five main groups of the clades could be found.

**Figure 4:**
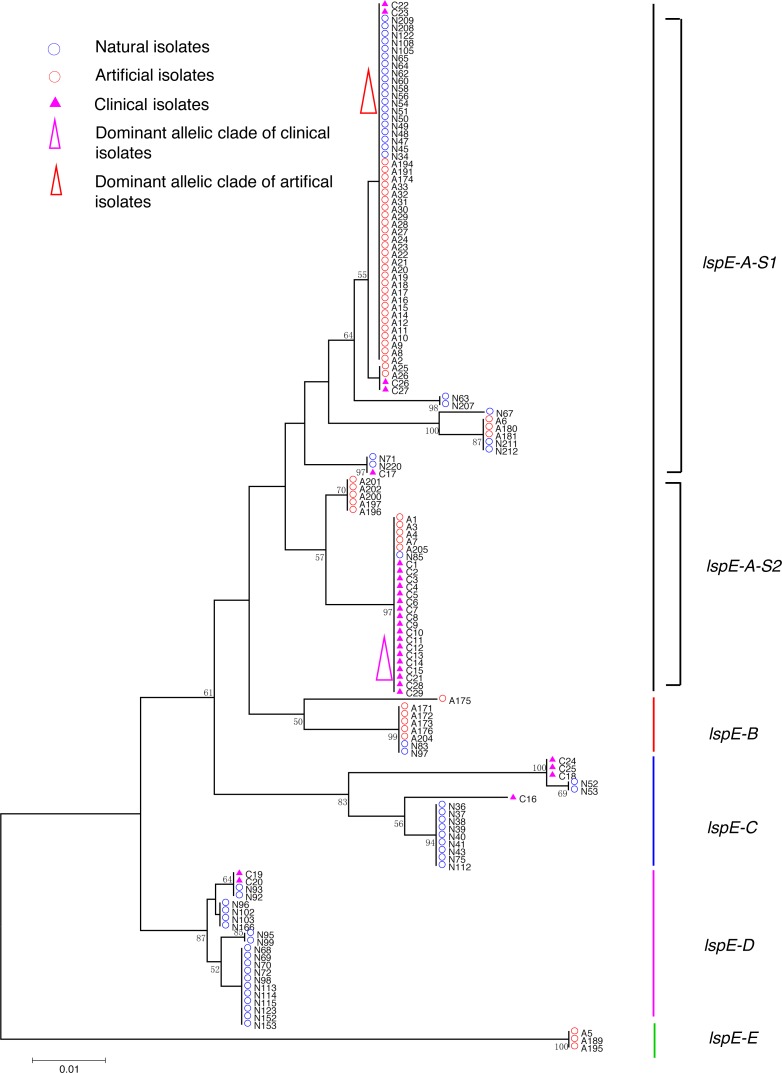
Neighbor-Joining tree of *L. pneumophila* isolates from DNA sequences of *lspE* locus. Strain types, source natures and geographic locations of these isolates were shown in Table S1. Bootstrap support values (1,000 replicates) for nodes higher than 50% are indicated next to the corresponding node. Five main groups of the clades could be found.

**Figure 5:**
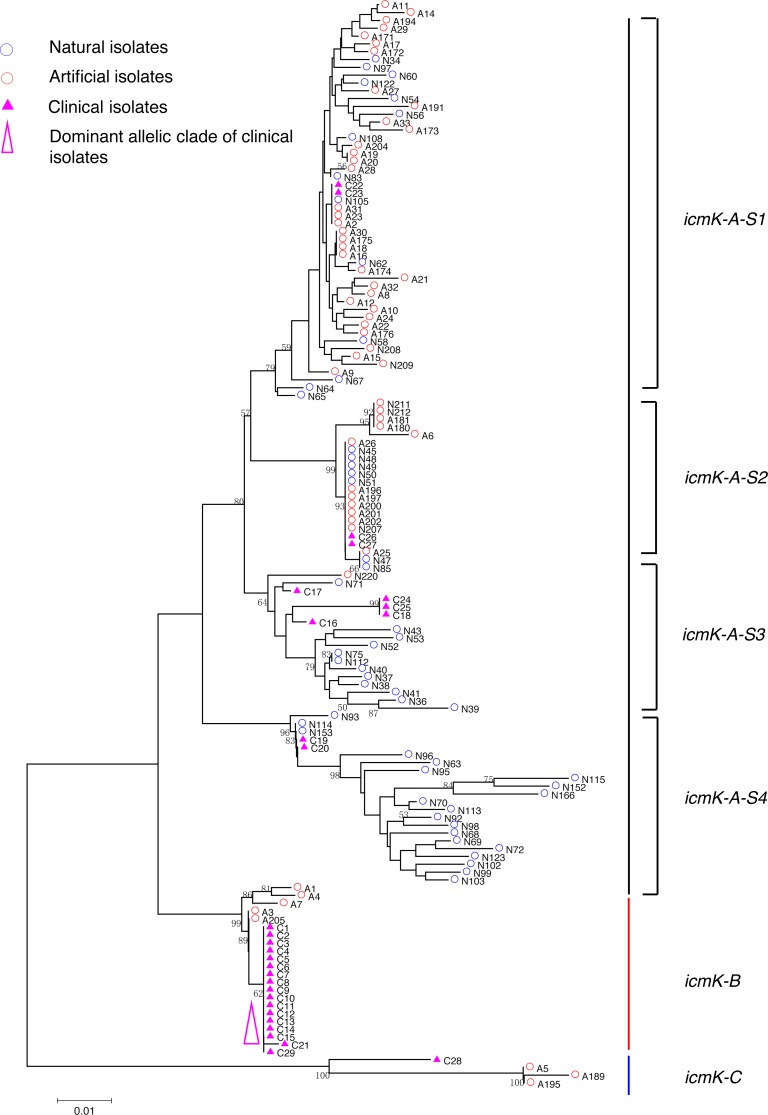
Neighbor-Joining tree of *L. pneumophila* isolates from DNA sequences of *icmK* locus. Strain types, source natures and geographic locations of these isolates were shown in Table S1. Bootstrap support values (1,000 replicates) for nodes higher than 50% are indicated next to the corresponding node. Three main groups of the clades could be found.

### Phylogenetic analysis of the five gene loci

Phylogenetic trees were derived separately for each locus to test the phylogenetic relationships between these isolates. The NJ trees showed three to six main groups (Figs. 1 to 5). The number of haplotypes in these loci ranged from 15 (*lssD* locus) to 89 (*icmK* locus). The NV loci displayed a similar number of haplotypes (23 for *cca* and 22 for *trpA*), while the virulence loci displayed large variable range of haplotypes (16 for *lssD*, 19 for *lsp*E and 89 for *icmK*). More than half of the clinical isolates (16/29 to 18/29, 55.17% to 62.07%) presented a single dominant allelic profile in the five loci (Figs. 1 to 5). Similarly, more than half (27/51 to 38/51, 52.94% to 74.51%) of the artificial isolates presented a distinct dominant allelic profile of the four loci (Figs. 1 to 4). Some of the allelic profiles were unique to clinical, artificial or natural isolates. Six allelic profiles of *cca* were unique to clinical isolates, comprising 26.09% of all profiles. Two to six allelic profiles of *trpA, lssD*, *lspE* and *icmK* loci were unique to clinical isolates, constituting 6.74% to 25% of the select profiles. A significantly different proportion of unique alleles of the five loci between natural and artificial strains was found (*P* = 0.0008, paired *t*-test). 30.43% (7/23), 40.91% (9/22), 40% (6/15), 42.11% (8/19), and 50.56% (45/89) allelic profiles of the *cca*, *trpA, lssD*, *lspE* and *icmK* loci were unique to natural isolates, respectively. By contrast, only 4.35% (1/23), 13.64% (3/22), 6.67% (1/15), 15.79% (3/19), and 35.96% (32/89) profiles of the *cca*, *trpA, lssD*, *lspE* and *icmK* loci were unique to artificial isolates. Most of the clinical isolates distributed in group A cluster of *cca* locus (86.21%, 25/29) and mostly in the subgroup 2 (cca-A-S2, 68.97%, 20/29); group B cluster of *trpA* locus (68.97%, 20/29) and mostly in the subgroup 2 (trpA-B-S2, 55.17%, 16/29); group B cluster of *lssD* locus (58.62%, 17/29), group A cluster of *lspE* locus (79.31%, 23/29) and mostly in the subgroup 2 (lspE-A-S2, 62.07%, 18/29); group B cluster of *icmK* locus (58.62%, 17/29). Artificial environmental isolates mainly distributed in group B cluster of *cca* locus (64.71%, 33/51), group A cluster of *trpA* locus (70.59%, 36/51*),* group A clusters of *lssD* locus (76.47%, 39/51), group A clusters of *lspE* (82.35%, 42/51) and group A cluster of *icmK* (84.31%, 43/51) (Figs. 1 to 5). The natural isolates in the NJ trees of *cca*, *trpA*, *lssD*, and *lspE* were more dispersed, while in the NJ tree of *icmK*, there mainly distributed in the group A, subgroup 3 and 4 clusters (Fig. 5).

### Relationships between clinical, natural and artificial isolates

We performed a hierarchical analysis of molecular variance (AMOVA) for the 139 isolates based on five loci considered above. The largest proportion of the genetic variation in each locus was found within populations, as this level accounted for 76.39% to 90.88% of the total variation (Table 2). The proportion of the total genetic variation explained by differences between clinical, artificial and natural isolates was small, ranging from −5.04% for *lssD* to 18.87% for *cca*, and it was not significant (Table 2). In contrast, geographical difference contributed to a part of genetic variation, especially for the virulence loci (accounting for 10.38% to 21.78% variation), and was all significant.

**Table 2:** Analysis of molecular variance of each locus.

Gene locus	Source of variation	*d.f.*	Sum of squares	Variance components	Percentage of variation	*F*-statistics
*cca*	Among groups	2	259.278	2.49795 Va	18.67	*FCT* = 0.18668
	Among populations within groups	3	42.796	0.25207 Vb	1.88	*FSC* = 0.02316
	Within populations	133	1413.883	10.63070 Vc	79.45	*FST* = 0.20552^*^
	Total		1715.957	13.38072		
*trpA*	Among groups	2	66.418	0.47727 Va	9.73	*FCT* = 0.05807
	Among populations within groups	3	23.622	0.25700 Vb	5.24	*FSC* = 0.14977^*^
	Within populations	133	554.392	4.16836 Vc	85.02	*FST* = 0.09735^*^
	Total		644.432	4.90263		
*lssD*	Among groups	2	48.770	−0.41909 Va	−5.04	*FCT* = − 0.05043
	Among populations within groups	3	73.572	1.17713 Vb	14.17	*FSC* = 0.13486^*^
	Within populations	133	1004.356	7.55155 Vc	90.88	*FST* = 0.09123^*^
	Total		1126.698	8.30959		
*lspE*	Among groups	2	85.132	−0.08745 Va	−1.39	*FCT* = − 0.01387
	Among populations within groups	3	74.467	1.37339 Vb	21.78	*FSC* = 0.21481^*^
	Within populations	133	667.660	5.02000 Vc	79.61	*FST* = 0.20393^*^
	Total		827.259	6.30594		
*icmK*	Among groups	2	198.660	1.34135 Va	13.23	*FCT* = 0.13230
	Among populations within groups	3	68.765	1.05258 Vb	10.38	*FSC* = 0.11964^*^
	Within populations	133	1030.086	7.74501 Vc	76.39	*FST* = 0.23611^*^
	Total		1297.511	10.13893		

**Notes.**

* indicates *P* < 0.05.

### Evolution and recombination analysis

**Figure 6:**
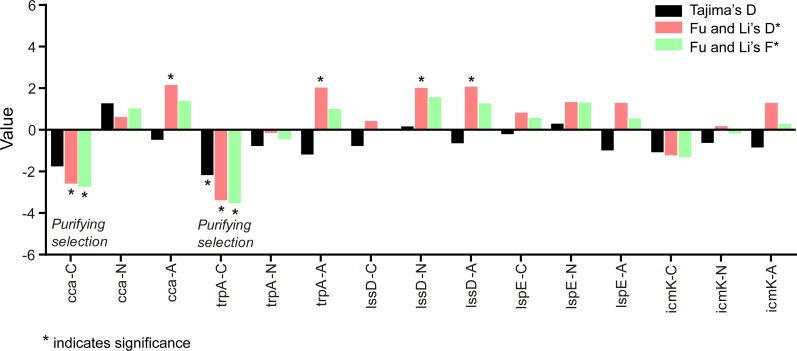
Tajima’s D, Fu and Li’s D* and F* test for the five gene loci of *L. pneumophila* from different sources. C indicates clinical isolates, N indicates natural isolates, and A indicates artificial isolates.

Tajima’s D, Fu and Li’s D* and F* statistics were calculated for testing the mutation neutrality hypothesis. The results showed most of the genes were in accord with the neutral hypothesis, except the NV genes of clinical isolates (Fig. 6, Table 3).

**Table 3:** Summary of neutrality for the five gene loci in *L. pneumophila* clinical (C), artificial (A), and natural (N) isolates.

Gene type	Locus	Strain type	Tajima’s D	Fu and Li’s D* test	Fu and Li’s F* test	
NV genes	*cca*	C	−1.75425, 0.10>*P*>0.05	**−2.57998**, ***P*** < **0.05**	**−2.72586**, ***P*** < **0.05**	**Purifying selection**
		N	1.27280, *P* > 0.10	0.61870, *P* > 0.10	1.03831, *P* > 0.10	Neutral
		A	−0.47005, *P* > 0.10	**2.15319**, ***P*** < **0.02**	1.39413, *P* > 0.10	Neutral
	*trpA*	C	**−2.17325**, ***P*** < **0.01**	**−3.38643**, ***P*** < **0.02**	**−3.52727**, ***P*** < **0.02**	**Purifying selection**
		N	−0.77493, *P* > 0.10	−0.13862, *P* > 0.10	−0.44983, *P* > 0.10	Neutral
		A	−1.18806, *P* > 0.10	**2.03417**, ***P*** < **0.02**	1.00976, *P* > 0.10	Neutral
Virulence genes	*lssD*	C	−0.77832, *P* > 0.10	0.42930, *P* > 0.10	0.03804, *P* > 0.10	Neutral
		N	0.16541, *P* > 0.10	**2.01583**, ***P*** < **0.02**	1.56550, 0.05<*P*<0.10	Neutral
		A	−0.64072, *P* > 0.10	**2.07816**, ***P*** < **0.02**	1.27560, *P* > 0.10	Neutral
	*lspE*	C	−0.20029, *P* > 0.10	0.82007, *P* > 0.10	0.57579, *P* > 0.10	Neutral
		N	0.29705, *P* > 0.10	1.33893, *P* > 0.10	1.31199, *P* > 0.10	Neutral
		A	−0.98456, *P* > 0.10	1.29642, *P* > 0.10	0.54402, *P* > 0.10	Neutral
	*icmK*	C	−1.07929, *P* > 0.10	−1.12272, *P* > 0.10	−1.30880, *P* > 0.10	Neutral
		N	−0.62353, *P* > 0.10	0.17035, *P* > 0.10	−0.16288, *P* > 0.10	Neutral
		A	−0.84707, *P* > 0.10	1.29035, *P* > 0.10	0.58803, *P* > 0.10	Neutral

The intragenic recombination events of each gene locus in different types of isolates were detected using RDP4 individually. For clinical isolates, RDP reported recombination events happened in the *cca* locus of C17, C21, C22, and C23, in the *lssD* locus of C17, and in the *lspE* locus of C16. No recombination event was identified on these loci of artificial environmental isolates. However, recombination events were detected only in virulence loci of natural isolates including the *lssD* locus of N71 and N220, the *lspE* locus of N52 and N53 and the *icmK* locus of N115 and N152. Details are shown in Table 4. These results might indicate that horizontal exchange of genetic material of virulence genes in clinical and natural isolates was more widespread than that in artificial isolates, and it was more prevalent in natural isolates.

**Table 4:** Intragenic recombination detection of the five loci in Clinical (C), artificial (A), and natural (N) isolates by using six different methods implemented in RDP software.

Gene type	Locus	Strain type^g^	Recombination events	Recombinant isolates	Major parent^a^	Minor parent^b^	Detection methods implemented in RDP software^c^					
							RDP	GENECONV	Bootscan	Maxchi	Chimaera	SiSscan
NV genes	*cca*	C	1	C17	C2	C23^d^	N^e^	N	N	Y^f^	Y	Y
			2	C21, C22, C23	C27^d^	C24	N	Y	N	Y	Y	Y
Virulence genes	*lssD*	C	1	C17	C28	C16	Y	Y	Y	Y	Y	Y
		N	1	N71, N220	N67	N93^d^	N	N	Y	Y	Y	Y
	*lspE*	C	1	C16	C25	C29	N	N	N	Y	Y	Y
		N	1	N52, N53	N112	N67^d^	N	N	N	Y	Y	Y
	*icmK*	N	1	N115, N152	N43^d^	N113	N	N	N	Y	Y	Y

**Notes.**

a Major parent: parent contributing the larger fraction of sequence.

b Minor parent: parent ST contributing the smaller fraction of sequence.

c Recombination events detected by more than two methods were shown.

d Sequences of the strain was used to infer the existence of a missing parental sequence.

e N indicates non-significant results.

f Y indicates significant results with *P* < 0.01.

g No recombination event was detected in the five loci of artificial isolates.

## Discussion

Although whole-genome sequencing (WGS) is a more informative technology to study genetic differences between different *L. pneumophila* isolates (Borges et al., 2016; Qin et al., 2016), investigations on special genes still provide clues in understanding *L. pneumophila* pathogenic mechanisms and epidemiological characteristics (Costa et al., 2012; Costa et al., 2014; Costa et al., 2010). Many studies have compared the genetic differences of *L.* *pneumophila* isolates from different sources, but these studies paid less attention to NV genes (Costa et al., 2014; Ko et al., 2003). Therefore, we have compared genetic variability of clinical, artificial and natural isolates of *L. pneumophila* at the nucleotide level in five coding loci, including two NV genes and three key virulence genes. These loci were researched as representative NV genes and virulence genes in this study. In addition to the two NV genes we studied, three NV genes including *rpoB*, DNA topoisomerase I gene and DNA polymerase III subunits gamma gene were also included in the study as our initial plan. However, alignment of the sequences from the NCBI database showed a very small variability (<10% sequence variation, Table S4) of these genes within *L. pneumophila* strains. We supposed that they might not provide sufficient resolution in determining the genetic difference between isolates from different source. Thus, they were not included in the following study. Globally representative clinical isolates were included in this study and compared with the environmental isolates due to the lack of *L. pneumophila* clinical isolates in China. For the NV genes, most of the genetic diversity parameters (*π* for *cca* and S, *θ*, *η*; for both *cca* and *trpA*) that were not directly dependent on sample size, were higher in the clinical isolates. In contrast, genetic diversity parameters of virulence loci (*π*, S, k of the three loci; and *θ* of *lssD*, *icmK* loci) were higher in the natural isolates. These results suggested that different evolutionary patterns existed between virulence genes and NV genes. It is well believed that environmental protozoa inhabiting the natural water sources, provided the primary evolutionary pressure for *Legionella* to obtain and maintain virulence factors (Moliner, Fournier & Raoult, 2010; Richards et al., 2013) and lead to a relatively higher genetic diversity in virulence genes. This was supported by our study. (Table 1). Similar ratios of *dN/dS* were found in the same loci of *L. pneumophila* isolates from different sources. Low ratios of *dN/dS* in both NV loci and virulence loci indicated these loci might be under purifying selection (Sobrinho Jr & De Brito, 2012). In this case, genetic variation occurs when it does not confer a significant disadvantage on surviving variant, and *dN/dS* ratios reflect general restrictions on gene and protein variability (Costa et al., 2014).

NJ trees showed that some alleles (including NV and virulence loci) were restricted to the clinical isolates although most of them were shared with both the clinical and environmental isolates. More than half of the clinical isolates and artificial isolates presented a single dominant allele of *cca, trpA, lssD*, *lspE* (Figs. 1 to 4)*.* This result supported the hypothesis proposed by Coscolla that clinical isolates were a small specific subset of all genotypes existing in nature, perhaps representing an especially adapted group of clones (Coscolla & Gonzalez-Candelas, 2009). We could also conclude that artificial isolates were also a non-random subset of all genotypes existing in nature, and only those more adaptive could inhabit an artificial environment. Some alleles were natural, artificial or clinical isolates tropic, indicating that isolates with these alleles were better-adapted in the selected environment. The relatively small proportion of unique allelic profiles in *cca, trpA, lssD* and *lspE* loci of artificial isolates further illustrated the intermediate post role of the artificial environment. Four *L. pneu mophila* isolates (A5, A189, A195, and C28) were more likely to experience a much more complex evolutionary history of *cca, trpA*, and *icmK* genes, because they were situated on their own distinct clade, separated from other isolates (Figs. 1, 2 and 5), envisioning the existence of several evolutionary reticulated events acting on these isolates (Costa et al., 2012). We found more allelic profiles the *icmK* locus (139 isolates possessed 89 allelic profiles, Fig. 5). This result indicated that *icmK* might be a suitable candidate locus for improving discrimination level in sequence-based epidemiological typing scheme of *L. pneumophila*. AMOVA results showed small differences among clinical, natural and artificial isolates, while a relatively larger genetic variation was found in the *cca* and *icmK* loci although the FCT values were not significant. Significant values of FST were found in all cases, supporting that some genetic differentiation (in specific genes) might exist between clinical and environmental isolates (Table 2).

Recombination events tend to happen in the genes with genetic plasticity, such as virulence genes (Coscolla & Gonzalez-Candelas, 2007; Gomez-Valero et al., 2011; Sánchez-Busó et al., 2014). These events are important for increasing *L. pneumophila* genetic pool by allowing the selection of new allelic patterns with increasing fitness or in a more neutral perspective (Zawierta et al., 2007). We observed recombination events in the three virulence loci and they mostly (6/8, 75%) happened in natural environmental isolates. We also found recombination events in the *cca* locus, but they all happened in clinical isolates. Neutrality tests showed that variations in the virulence genes of clinical and environmental isolates were under neutral evolution, indicating that the given virulence genes were probably implicated in conserved virulence mechanisms. This result was partly in accordance with Costa’s report that *lspE* locus maintained neutral evolution (Costa et al., 2012). Nevertheless, *trpA* and *cca* of clinical isolates showed significantly negative values of Tajima’s D, Fu and Li’s D* and F*. This could be interpreted as the presence of negative selection, an increased population size or subpopulation structure (Hughes, 2008; Jukes, 2000; Nei, Suzuki & Nozawa, 2010). These results also suggested that different evolutionary patterns existed between NV genes and virulence genes of *L. pneumophila* strains from different sources.

## Conclusions

In sum, we have characterized the genetic variability of two NV genes and three virulence genes in clinical, natural and artificial isolates of *L. pneumophila*. The results unveiled different genetic variability between NV genes and virulence genes in *L. pneumophila* from the clinical, artificial and natural sources. Recombination events played an important role in the molecular evolution of the NV genes of clinical isolates and the virulence genes of natural isolates, and might lead to the change of population structure of this bacterium. This work provides clues for future work on population-level and genetics-level questions about ecology and molecular evolution of *L. pneumophila,* as well as the genetic differences of NV genes and virulence genes between this host-range pathogen with different lifestyles.

##  Supplemental Information

10.7717/peerj.4114/supp-1 Table S1 *L. pneumophila* isolates information

10.7717/peerj.4114/supp-2 Table S2 Sequence variation in genes of *L. pneumophila* strains

10.7717/peerj.4114/supp-3 Table S3 Summary of *dS* and *dN* values of the genes in different types of *L. pneumophila* isolates

10.7717/peerj.4114/supp-4 Table S4 Sequence variation in *rpoB*, *DNA topoisomerase I , and DNA polymerase III subunits gamma* genes of *L. pneumophila* strains**

10.7717/peerj.4114/supp-5 Supplemental Information 1 Sequences of the five genes

## Additional Information and Declarations

### DNA Deposition

The following information was supplied regarding the deposition of DNA sequences: The 550 sequences from L. pneumophila environmental isolates determined in this study were deposited in the GenBank Nucleotide Sequence Database under accession numbers KY708328 –KY708437 (cca), KY708438 –KY708547 (trpA), KY708768 –KY708877 (lssD), KY708658 –KY708767 (lspE) and KY708548 –KY708657 (icmK). Sequence data can also be found in the Supplemental Information.
