# Supplementary material for: Molecular evolution of virulence genes and non-virulence genes in clinical, natural and artificial environmental Legionella pneumophila isolates
Source: PeerJ. 2017 Dec 4;5:e4114. doi: 10.7717/peerj.4114 (PMC5719964; doi:10.7717/peerj.4114)
Supplement: Table S1 [file peerj-05-4114-s001.docx]

**Table S1. *L. pneumophila* isolates information.**

| Strain designation | | Source nature | | | geographic location | collection date/ year | | ST type | | BioSample No. / (S*train name*) |
| --- | --- | --- | --- | --- | --- | --- | --- | --- | --- | --- |
| **^&^Clinical isolates (n = 29 )** | | | | | | | | | |  |
| C1 | N/A | | | United Kingdom | | | 2003-11-01 | | ST37 | SAMEA4535098 *(ST37)* |
| C2 | N/A | | | USA: Georgia | | | 2009-03-18 | | ST36 | SAMN05180032 *(C11_O)* |
| C3 | N/A | | | USA: Indiana | | | 1982-12-30 | | N/A | SAMN05180030 *(C9_S)* |
| C4 | N/A | | | USA: Colorado | | | 1993-03-12 | | N/A | SAMN05180029 *(C8_S)* |
| C5 | N/A | | | USA: New Jersey | | | 1996-01-22 | | N/A | SAMN05180027 *(C6_S)* |
| C6 | N/A | | | USA: Ohio | | | 1998-02-24 | | N/A | SAMN05180026 *(C5_P)* |
| C7 | N/A | | | USA: Georgia | | | 2000-03-20 | | N/A | SAMN05180025 *(C4_S)* |
| C8 | N/A | | | USA: Texas | | | 2006-06-07 | | N/A | SAMN05180024 *(C3_O)* |
| C9 | N/A | | | USA: Illinois | | | 2007-11-06 | | N/A | SAMN05179997 *(C2_S)* |
| C10 | N/A | | | USA: Pennsylvania | | | 1977-01-16 | | N/A | SAMN05180046 *(Philadelphia_3)* |
| C11 | N/A | | | USA: Pennsylvania | | | 1977-01-11 | | N/A | SAMN05180045 *(Philadelphia_2)* |
| C12 | N/A | | | USA: Pennsylvania | | | 1977-01-11 | | N/A | SAMN05180044 *(Philadelphia_1)* |
| C13 | N/A | | | Australia | | | 2000-05-01 | | N/A | SAMEA4067783 *(Lpm7613)* |
| C14 | N/A | | | Canada | | | N/A | | N/A | SAMN02603729 *(Thunder Bay)* |
| C15 | Lung tissue | | | USA: Denver | | | 1987 | | N/A | SAMN02603182 *(ATCC43290)* |
| C16 | N/A | | | Germany | | | 1999-01-01 | | ST42 | SAMEA4535099 *(ERS1434278)* |
| C17 | N/A | | | France: Lorraine | | | N/A | | N/A | SAMEA3138425 *(Lorraine)* |
| C18 | N/A | | | France: Lens | | | 2003 | | N/A | SAMEA3138253 *(Lens)* |
| C19 | N/A | | | Spain: Alcoy | | | 1999 | | N/A | SAMN02604292 *(2300/99 Alcoy)* |
| C20 | N/A | | | United Kingdom | | | N/A | | N/A | SAMN02603241 *(Corby)* |
| C21 | N/A | | | USA: Nebraska | | | 1990-06-07 | | N/A | SAMN05180031 *(C10_S)* |
| C22 | N/A | | | USA | | | 1947 | | N/A | SAMN05198688 *(OLDA)* |
| C23 | N/A | | | France: Paris | | | 1987 | | N/A | SAMEA3138252 *(Paris)* |
| C24 | N/A | | | [Norway](https://www.ncbi.nlm.nih.gov/biosample?term=) | | | 2008 | | N/A | SAMN05513578 *(FFI104)* |
| C25 | N/A | | | Norway | | | 2005 | | N/A | SAMN05513576 *(FFI102)* |
| C26 | N/A | | | USA: New York | | | 2012 | | N/A | SAMN04634513 *(D-7630)* |
| C27 | N/A | | | USA: New York | | | 2012 | | N/A | SAMN04633978 *(D-7632)* |
| C28 | Sputum | | | USA: Pennsylvania | | | 2012 | | N/A | SAMN04438273 *(D-7119)* |
| C29 | N/A | | | USA: Deleware | | | 1994-08-10 | | N/A | SAMN05180028 *(C7_O)* |
| **Artificial isolates (n = 51)** | | | | | | | | | |  |
| A1 | ACC# | | | Jiangmen: Tianyue hotel | | | 2004-07-30 | | 630 | N/A |
| A2 | ACC | | | Jiangmen: Tianyue hotel | | | 2004-07-30 | | 1 | N/A |
| A3 | ACC | | | Jiangmen: Tianyue hotel | | | 2004-07-30 | | 242 | N/A |
| A4 | ACC | | | Jiangmen: Tianyue hotel | | | 2004-07-30 | | 242 | N/A |
| A5 | ACC | | | Jiangmen: Tianyue hotel | | | 2004-07-30 | | 1778 | N/A |
| A6 | ACC | | | Jiangmen: Tianyue hotel | | | 2004-07-30 | | 1417 | N/A |
| A7 | ACC | | | Jiangmen: Tianyue hotel | | | 2004-07-30 | | 242 | N/A |
| A8 | ACC | | | Jiangmen: Tianyue hotel | | | 2006-06-15 | | 1 | N/A |
| A9 | ACC | | | Jiangmen: Tianyue hotel | | | 2006-06-15 | | 1 | N/A |
| A10 | ACC | | | Jiangmen: Tianyue hotel | | | 2006-06-15 | | 1 | N/A |
| A11 | ACC | | | Jiangmen: Tianyue hotel | | | 2006-06-15 | | 1 | N/A |
| A12 | ACC | | | Jiangmen: Tianyue hotel | | | 2006-06-15 | | 1 | N/A |
| A14 | ACC | | | Jiangmen: Tianyue hotel | | | 2006-06-15 | | 1 | N/A |
| A15 | ACC | | | Jiangmen: Tianyue hotel | | | 2006-06-15 | | 1048 | N/A |
| A16 | ACC | | | Jiangmen: Tianyue hotel | | | 2006-06-15 | | 1 | N/A |
| A17 | ACC | | | Jiangmen: Tianyue hotel | | | 2006-06-15 | | 1 | N/A |
| A18 | ACC | | | Jiangmen: Tianyue hotel | | | 2006-06-15 | | 1 | N/A |
| A19 | ACC | | | Jiangmen: Tianyue hotel | | | 2006-06-15 | | 1 | N/A |
| A20 | ACC | | | Jiangmen: Tianyue hotel | | | 2006-06-15 | | 1 | N/A |
| A21 | ACC | | | Guangzhou: Zijinyuan hotel | | | 2003-10-01 | | 1 | N/A |
| A22 | ACC | | | Guangzhou: Zijinyuan hotel | | | 2003-10-01 | | 1 | N/A |
| A23 | ACC | | | Guangzhou: Zijinyuan hotel | | | 2003-10-01 | | 630 | N/A |
| A24 | ACC | | | Guangzhou: Zijinyuan hotel | | | 2003-10-01 | | 1 | N/A |
| A25 | ACC | | | Guangzhou: Zijinyuan hotel | | | 2003-10-01 | | 59 | N/A |
| A26 | ACC | | | Guangzhou: Zijinyuan hotel | | | 2003-10-01 | | 59 | N/A |
| A27 | ACC | | | Guangzhou: Zijinyuan hotel | | | 2003-10-01 | | 1262 | N/A |
| A28 | ACC | | | Guangzhou: Zijinyuan hotel | | | 2003-10-08 | | 1040 | N/A |
| A29 | ACC | | | Guangzhou: Zijinyuan hotel | | | 2003-10-08 | | 1046 | N/A |
| A30 | ACC | | | Guangzhou: Zijinyuan hotel | | | 2003-10-08 | | 1 | N/A |
| A31 | ACC | | | Guangzhou: Zijinyuan hotel | | | 2003-10-08 | | 1 | N/A |
| A32 | ACC | | | Guangzhou: Zijinyuan hotel | | | 2003-10-08 | | 172 | N/A |
| A33 | ACC | | | Guangzhou: Zijinyuan hotel | | | 2003-10-08 | | 1 | N/A |
| A171 | ACC | | | Jiangmen: Xinhui Yinbing Hotel | | | 2007-09-01 | | 752 | N/A |
| A172 | ACC | | | Jiangmen: Xinhui Yinbing Hotel | | | 2007-09-01 | | 752 | N/A |
| A173 | ACC | | | Jiangmen: Xinhui Yinbing Hotel | | | 2007-09-01 | | 752 | N/A |
| A174 | ACC | | | Jiangmen: Xinhui Yinbing Hotel | | | 2007-09-01 | | 1777 | N/A |
| A175 | ACC | | | Jiangmen: Xinhui Yinbing Hotel | | | 2007-09-01 | | 752 | N/A |
| A176 | ACC | | | Jiangmen: Xinhui Yinbing Hotel | | | 2007-09-01 | | 752 | N/A |
| A180 | ACC | | | Jiangmen: Xinhui Yinbing Hotel | | | 2007-09-01 | | 1417 | N/A |
| A181 | ACC | | | Jiangmen: Xinhui Yinbing Hotel | | | 2007-09-01 | | 1417 | N/A |
| A189 | ACC | | | Jiangmen: Xinhui Gangzhou Hotel | | | 2007-09-01 | | 160 | N/A |
| A191 | ACC | | | Jiangmen: Xinhui Gangzhou Hotel | | | 2007-09-01 | | 1 | N/A |
| A194 | ACC | | | Jiangmen: Xinhui Gangzhou Hotel | | | 2007-09-01 | | 1 | N/A |
| A195 | ACC | | | Jiangmen: Xinhui Gangzhou Hotel | | | 2007-09-01 | | 1779 | N/A |
| A196 | ACC | | | Jiangmen: Xinhui Gangzhou Hotel | | | 2007-09-01 | | 1054 | N/A |
| A197 | ACC | | | Jiangmen: Xinhui Gangzhou Hotel | | | 2007-09-01 | | 1054 | N/A |
| A200 | ACC | | | Jiangmen: Xinhui Gangzhou Hotel | | | 2007-09-01 | | 1054 | N/A |
| A201 | ACC | | | Jiangmen: Xinhui Gangzhou Hotel | | | 2007-09-01 | | 752 | N/A |
| A202 | ACC | | | Jiangmen: Xinhui Gangzhou Hotel | | | 2007-09-01 | | 1054 | N/A |
| A204 | ACC | | | Jiangmen: Xinhui Yujing Hotel | | | 2007-09-01 | | 752 | N/A |
| A205 | ACC | | | Jiangmen: Xinhui Yujing Hotel | | | 2007-09-01 | | 93 | N/A |
| **Natural isolates (n = 59)** | | | | | | | | | | |
| N34 | Pond | | Guangzhou: Huangpu village | | | | 2006-08-01 | | 242 | N/A |
| N36 | Pond | | Guangzhou: Huangpu village | | | | 2006-08-01 | | 739 | N/A |
| N37 | Pond | | Guangzhou: Huangpu village | | | | 2006-08-01 | | 739 | N/A |
| N38 | Pond | | Guangzhou: Huangpu village | | | | 2006-08-01 | | 739 | N/A |
| N39 | Pond | | Guangzhou: Huangpu village | | | | 2006-08-01 | | 739 | N/A |
| N40 | Pond | | Guangzhou: Huangpu village | | | | 2006-08-01 | | 739 | N/A |
| N41 | Pond | | Guangzhou: Huangpu village | | | | 2006-08-01 | | 739 | N/A |
| N43 | Pond | | Guangzhou: Huangpu village | | | | 2006-08-01 | | 739 | N/A |
| N45 | Pond | | Guangzhou: Huangpu village | | | | 2006-08-01 | | 1267 | N/A |
| N47 | Pond | | Guangzhou: Huangpu village | | | | 2006-08-01 | | 1267 | N/A |
| N48 | Pond | | Guangzhou: Huangpu village | | | | 2006-08-01 | | 1267 | N/A |
| N49 | Pond | | Guangzhou: Huangpu village | | | | 2006-08-01 | | 1267 | N/A |
| N50 | Pond | | Guangzhou: Huangpu village | | | | 2006-08-01 | | 1267 | N/A |
| N51 | Pond | | Guangzhou: Huangpu village | | | | 2006-08-01 | | 1267 | N/A |
| N52 | Pond | | Guangzhou: Huangpu village | | | | 2006-08-01 | | 1266 | N/A |
| N53 | Pond | | Guangzhou: Huangpu village | | | | 2006-08-01 | | 1266 | N/A |
| N54 | Lake | | Guangzhou: Liuhua park | | | | 2006-09-01 | | 630 | N/A |
| N56 | Lake | | Guangzhou: Liuhua park | | | | 2006-09-01 | | 630 | N/A |
| N58 | Lake | | Guangzhou: Liuhua park | | | | 2006-09-01 | | 630 | N/A |
| N60 | Lake | | Guangzhou: Liuhua park | | | | 2006-09-01 | | 630 | N/A |
| N62 | Lake | | Guangzhou: Liuhua park | | | | 2006-09-01 | | 1048 | N/A |
| N63 | Lake | | Guangzhou: Liuhua park | | | | 2006-09-01 | | 1782 | N/A |
| N64 | Lake | | Guangzhou: Liuhua park | | | | 2006-09-01 | | 1785 | N/A |
| N65 | Lake | | Guangzhou: Liuhua park | | | | 2006-09-01 | | 1785 | N/A |
| N67 | Lake | | Guangzhou: Liuhua park | | | | 2006-09-01 | | 1788 | N/A |
| N68 | Lake | | Guangzhou: Liuhua park | | | | 2006-09-01 | | 1048 | N/A |
| N69 | Lake | | Guangzhou: Liuhua park | | | | 2006-09-01 | | 1048 | N/A |
| N70 | Lake | | Guangzhou: Liuhua park | | | | 2006-09-01 | | 1048 | N/A |
| N71 | Lake | | Guangzhou: Liuhua park | | | | 2006-09-01 | | 45 | N/A |
| N72 | Lake | | Guangzhou: Liuhua park | | | | 2006-09-01 | | 1048 | N/A |
| N75 | Lake | | Guangzhou: Liuhua park | | | | 2006-09-01 | | 1049 | N/A |
| N83 | Lake | | Guangzhou: Liuhua park | | | | 2006-09-01 | | 752 | N/A |
| N85 | Lake | | Guangzhou: Liuhua park | | | | 2006-09-01 | | 114 | N/A |
| N92 | Lake | | Guangzhou: Liuhua park | | | | 2006-09-01 | | 1051 | N/A |
| N93 | Lake | | Guangzhou: Liuhua park | | | | 2006-09-01 | | 1050 | N/A |
| N95 | Lake | | Guangzhou: Liuhua park | | | | 2006-09-01 | | 1052 | N/A |
| N96 | Lake | | Guangzhou: Liuhua park | | | | 2006-09-01 | | 1053 | N/A |
| N97 | Lake | | Guangzhou: Liuhua park | | | | 2006-09-01 | | 752 | N/A |
| N98 | Lake | | Guangzhou: Liuhua park | | | | 2006-09-01 | | 1048 | N/A |
| N99 | Lake | | Guangzhou: Liuhua park | | | | 2006-09-01 | | 1052 | N/A |
| N102 | Lake | | Guangzhou: Liuhua park | | | | 2006-09-01 | | 1053 | N/A |
| N103 | Lake | | Guangzhou: Liuhua park | | | | 2006-09-01 | | 1053 | N/A |
| N105 | Lake | | Guangzhou: Tianhe park | | | | 2006-09-01 | | 1263 | N/A |
| N108 | Lake | | Guangzhou: Tianhe park | | | | 2006-09-01 | | 1777 | N/A |
| N112 | Lake | | Guangzhou: Tianhe park | | | | 2006-09-01 | | 1049 | N/A |
| N113 | Lake | | Guangzhou: Tianhe park | | | | 2006-09-01 | | 1048 | N/A |
| N114 | Lake | | Guangzhou: Tianhe park | | | | 2006-09-01 | | 1048 | N/A |
| N115 | Lake | | Guangzhou: Tianhe park | | | | 2006-09-01 | | 1048 | N/A |
| N122 | Lake | | Guangzhou: Yuexiu park | | | | 2006-09-01 | | 1777 | N/A |
| N123 | Lake | | Guangzhou: Yuexiu park | | | | 2006-09-01 | | 1048 | N/A |
| N152 | Lake | | Guangzhou: Nanhu | | | | 2006-11-01 | | 1048 | N/A |
| N153 | Lake | | Guangzhou: Nanhu | | | | 2006-11-01 | | 1048 | N/A |
| N166 | Lake | | Guangzhou: Luhu | | | | 2006-11-01 | | 1048 | N/A |
| N207 | River | | Jiangmen: Xinhui Green park | | | | 2006-11-01 | | 1781 | N/A |
| N208 | River | | Jiangmen: Xinhui Green park | | | | 2006-11-01 | | 1 | N/A |
| N209 | River | | Jiangmen: Xinhui Green park | | | | 2007-10-01 | | 1 | N/A |
| N211 | River | | Jiangmen: Xinhui Gangzhou | | | | 2007-10-01 | | 1417 | N/A |
| N212 | River | | Jiangmen: Xinhui Gangzhou | | | | 2007-10-01 | | 1417 | N/A |
| N220 | River | | Jiangmen: Xinhui Jade lake | | | | 2007-10-01 | | 45 | N/A |

**^&^** Clinical isolates indicate those isolated from clinical samples or those were disease-related.

#ACC indicates air conditioning cooling tower water.

N/A indicates not available.
