# Supplementary material for: Molecular evolution of virulence genes and non-virulence genes in clinical, natural and artificial environmental Legionella pneumophila isolates
Source: PeerJ. 2017 Dec 4;5:e4114. doi: 10.7717/peerj.4114 (PMC5719964; doi:10.7717/peerj.4114)
Supplement: Table S2 [file peerj-05-4114-s002.docx]

**Table S2. Sequence variation in genes of *L. pneumophila* strains.**

| Gene | Primers (5’-3’) | Fragment size (bp)  of amplified  product | Size of region used to determine allele type | Region used for  allele assignment | *No. of variable  sites | % Sequence  variation |
| --- | --- | --- | --- | --- | --- | --- |
| *cca* | ATGAAAGTCTACTTGGTAGGC | 1197 | 1082 | 52-1133 | 138 | 12.75 |
|  | CTCTACACAAGCCACACG |  |  |  |  |  |
| *trpA* | ATGAACCGTATTGATAAGACTCTGG | 797 | 748 | 30-777 | 81 | 10.83 |
|  | TCGATAGCTTGCCTCATGGA |  |  |  |  |  |
| *lssD* | TCACGGATGAGAGAGTTGAATC | 385 | 330 | 439-768 | 84 | 25.45 |
|  | GCTTCAATTAATAAAGTATCATCAAGCG |  |  |  |  |  |
| *lspE* | ACTCTGTCTTCATAAGTTTCAATGTG | 380 | 331 | 66-396 | 70 | 21.15 |
|  | GCCAAAAGCAATGGAATTGTTG |  |  |  |  |  |
| *icmK* | GCTGATCAATCAGATGATGCTC | 436 | 385 | 105-489 | 112 | 29.09 |
|  | GTGATAATCTTATTACTGGTGGTG |  |  |  |  |  |

* Number of variable sites of the gene fragments were obtained by analyzing 45 (*cca*), 68 (*trpA*), 56 (*lssD*), 36 (*icmK*) ,and 87 (*lspE*) known sequences in NCBI database, using Dnasp v5 (http://www.ub.edu/dnasp/).
