# Supplementary material for: Molecular evolution of virulence genes and non-virulence genes in clinical, natural and artificial environmental Legionella pneumophila isolates
Source: PeerJ. 2017 Dec 4;5:e4114. doi: 10.7717/peerj.4114 (PMC5719964; doi:10.7717/peerj.4114)
Supplement: Table S3 [file peerj-05-4114-s003.docx]

| **Gene type** | **locus** | **Strain type** | ***dS*** | ***dN*** |
| --- | --- | --- | --- | --- |
| NV genes | *cca* | C^#^ | 0.0625 | 0.00646 |
|  |  | N^$^ | 0.0649 | 0.00704 |
|  |  | A^*^ | 0.0832 | 0.00719 |
|  | *trpA* | C | 0.0472 | 0.00299 |
|  |  | N | 0.0338 | 0.00338 |
|  |  | A | 0.0444 | 0.00356 |
| Virulence genes | *lssD* | C | 0.2076 | 0.01042 |
|  |  | N | 0.3091 | 0.01093 |
|  |  | A | 0.2470 | 0.00982 |
|  | *lspE* | C | 0.1471 | 0.00243 |
|  |  | N | 0.1749 | 0.00661 |
|  |  | A | 0.1501 | 0.00511 |
|  | *icmK* | C | 0.1250 | 0.01142 |
|  |  | N | 0.1160 | 0.02951 |
|  |  | A | 0.1244 | 0.01995 |

**Table S3.** **Summary of *dS* and *dN* values of the genes in different types of *L. pneumophila* isolates.**

# C indicates clinical isolates

$ N indicates natural environmental isolates

* A indicates artificial environmental isolates
