# Supplementary material for: Molecular evolution of virulence genes and non-virulence genes in clinical, natural and artificial environmental Legionella pneumophila isolates
Source: PeerJ. 2017 Dec 4;5:e4114. doi: 10.7717/peerj.4114 (PMC5719964; doi:10.7717/peerj.4114)
Supplement: Table S4 [file peerj-05-4114-s004.docx]

**Table S4. Sequence variation in *rpoB*, *DNA topoisomerase I, and DNA polymerase III subunits gamma* genes of *L. pneumophila* strains.**

| Gene | No. of analyzed sequences | Sequence length | *No. of variable  sites | % Sequence  variation |
| --- | --- | --- | --- | --- |
| *rpoB* | 62 | 4107 | 216 | 5.26 |
| *DNA topoisomerase I* | 51 | 2280 | 79 | 3.46 |
| *DNA polymerase*  *III subunits gamma* | 62 | 1671 | 135 | 8.08 |

* Number of variable sites of the gene were obtained by analyzing known sequences in NCBI database, using Dnasp v5 (http://www.ub.edu/dnasp/).
